
JOURNAL INFORMATION
==============================
NLM Title Abbreviation: Biospektrum (Heidelb)
Journal ID: BIOspektrum (Heidelb.)
NLM Journal ID: 9615685

Biospektrum

ISSN: 0947-0867
EISSN: 1868-6249

ARTICLE INFORMATION
==============================
PMCID: PMC7474491
DOI: 10.1007/s12268-020-1425-z
Article ID: 1425
Article version: 1
Subjects: Karriere, Köpfe & Konzepte

Buchrezension zu: Pest und Corona

Seifert Mathias Mathias.Seifert@hs-fresenius.de

grid.440934.e 0000 0004 0593 1824 Hochschule Fresenius, Idstein, Deutschland
Electronic publication date: 2020 Sep 5
Print publication date: 2020
Volume: 26
Issue: 5
First page: 578
Last page: 578
Copyright: © Der/die Autor(en) 2020
License: Open Access Dieser Artikel wird unter der Creative Commons Namensnennung 4.0 International Lizenz veröffentlicht, welche die Nutzung, Vervielfältigung, Bearbeitung, Verbreitung und Wiedergabe in jeglichem Medium und Format erlaubt, sofern Sie den/die ursprünglichen Autor(en) und die Quelle ordnungsgemäß nennen, einen Link zur Creative Commons Lizenz beifügen und angeben, ob Änderungen vorgenommen wurden.
Die in diesem Artikel enthaltenen Bilder und sonstiges Drittmaterial unterliegen ebenfalls der genannten Creative Commons Lizenz, sofern sich aus der Abbildungslegende nichts anderes ergibt. Sofern das betreffende Material nicht unter der genannten Creative Commons Lizenz steht und die betreffende Handlung nicht nach gesetzlichen Vorschriften erlaubt ist, ist für die oben aufgeführten Weiterverwendungen des Materials die Einwilligung des jeweiligen Rechteinhabers einzuholen.
Weitere Details zur Lizenz entnehmen Sie bitte der Lizenzinformation auf http://creativecommons.org/licenses/by/4.0/deed.de.
License URL: https://creativecommons.org/licenses/by/4.0/

issue-copyright-statement: © Springer-Verlag GmbH Deutschland, ein Teil von Springer Nature 2020

==============================
Funding Open Access funding provided by Project DEAL.


Literatur

Pest und Corona Pandemien in Geschichte, Gegenwart und Zukunft Heiner Fangerau und Alfons Labisch 192S., Herder, 2020. HC, 18,— €. ISBN: 9783451388798 Auch als EBook erhältlich
